# Trends in the awareness, acceptability, and usage of HIV pre-exposure prophylaxis among at-risk men who have sex with men in Toronto

**DOI:** 10.17269/s41997-018-0064-3

**Published:** 2018-04-26

**Authors:** Jayoti Rana, James Wilton, Shawn Fowler, Trevor A. Hart, Ahmed M. Bayoumi, Darrell H. S. Tan

**Affiliations:** 1 Centre for Urban Health Solutions, St. Michael’s Hospital, Toronto, Canada; 2 Ontario HIV Treatment Network, Toronto, Canada; 3 Hassle Free Clinic, Toronto, Canada; 4 Department of Psychology, Ryerson University, Toronto, Canada; 5 Dalla Lana School of Public Health, University of Toronto, Toronto, Canada; 6 Department of Medicine, University of Toronto, Toronto, Canada; 7 Department of Medicine, St. Michael’s Hospital, Toronto, Canada; 8 Institute of Health Policy, Management and Evaluation, University of Toronto, Toronto, Canada; 9 Division of Infectious Diseases, St. Michael’s Hospital, 30 Bond St, 4CC – Room 4-179, Toronto, ON M5B 1W8 Canada

**Keywords:** Pre-exposure prophylaxis, HIV, Men who have sex with men (MSM), HIV prevention, Prophylaxie pré-exposition, VIH, Hommes ayant des relations sexuelles avec des hommes (HARSAH), Prévention du VIH

## Abstract

**Objectives:**

Pre-exposure prophylaxis (PrEP) with daily oral tenofovir/emtricitabine dramatically reduces HIV risk in men who have sex with men (MSM). However, uptake is slow worldwide.

**Methods:**

We administered anonymous cross-sectional questionnaires to MSM presenting for anonymous HIV testing at a Toronto sexual health clinic at four successive time points during the period 2013–2016. We assessed trends in PrEP awareness, acceptability, and use over time using the Cochran-Armitage Trend Test, and identified barriers to using PrEP by constructing “PrEP cascades” using 2016 data. We assumed that to use PrEP, MSM must (a) be at risk for HIV, (b) be at objectively high risk (HIRI-MSM score ≥ 10), (c) perceive themselves to be at medium-to-high risk, (d) be aware of PrEP, (e) be willing to use PrEP, (f) have a family doctor, (g) be comfortable discussing sexual health with that doctor, and (h) have drug coverage/be willing to pay out of pocket.

**Results:**

MSM participants were mostly white (54–59.5%), with median age 31 years (IQR = 26–38). PrEP awareness and use increased significantly over time (both *p* < 0.0001), reaching 91.3% and 5.0%, respectively, in the most recent wave. Willingness to use PrEP rose to 56.5%, but this increase did not reach statistical significance (*p* = 0.06). The full cascade, ABCDEFGH, suggested few could readily use PrEP under current conditions (11/400 = 2.8%). The largest barriers, in descending order, were low self-perceived HIV risk, unwillingness to use PrEP, and access to PrEP providers.

**Conclusion:**

To maximize its potential public health benefits, PrEP scale-up strategies must address self-perceived HIV risk and increase access to PrEP providers.

## Résumé

**Objectifs:**

La prise quotidienne de ténofovir et d’emtricitabine à titre de prophylaxie pré-exposition (PPrE) orale réduit considérablement le risque de contracter le VIH chez les hommes ayant des relations sexuelles avec des hommes (HARSAH). L’adoption de cette PPrE à l’échelle mondiale est toutefois lente.

**Méthode:**

Nous avons fait passer des questionnaires transversaux anonymes à des HARSAH s’étant présentés pour un dépistage anonyme du VIH dans une clinique de santé sexuelle de Toronto à quatre moments consécutifs entre 2013 et 2016. Nous avons évalué les tendances liées à la connaissance, à l’acceptabilité et à l’utilisation de la PPrE au fil du temps à l’aide du test de tendance de Cochran-Armitage et déterminé les obstacles à l’utilisation de la PPrE en construisant des « cascades de PPrE » avec les données de 2016. Nous avons présumé que pour utiliser la PPrE, les HARSAH doivent: (a) être à risque de contracter le VIH, (b) courir un risque objectivement élevé (note ≥ 10 sur l’échelle HIRI-MSM), (c) se percevoir comme courant un risque moyen à élevé, (d) connaître la PPrE, (e) être disposés à utiliser la PPrE, (f) avoir un médecin de famille, (g) être à l’aise de parler de santé sexuelle avec ce médecin, et (h) avoir une assurance-médicaments ou être disposés à défrayer le coût des médicaments.

**Résultats:**

Les HARSAH participants étaient en majorité de race blanche (54% à 59,5%) et avaient un âge médian de 31 ans (écart interquartile 26–38). Leur connaissance et leur utilisation de la PPrE a sensiblement augmenté au fil du temps (*p* < 0,0001 dans les deux cas), pour atteindre 91,3% et 5% respectivement au cours du cycle le plus récent. Leur volonté d’utiliser la PPrE est passée à 56,5%, mais cette hausse n’était pas statistiquement significative (*p* = 0,06). La cascade complète, ABCDEFGH, montre que peu d’entre eux pouvaient facilement utiliser la PPrE dans leur situation actuelle (11/400 = 2,8%). Les plus grands obstacles, en ordre descendant, étaient le faible risque autoperçu de contracter le VIH, la réticence à utiliser la PPrE et le manque d’accès aux dispensateurs de la PPrE.

**Conclusion:**

Pour en maximiser les bienfaits éventuels pour la santé publique, les stratégies de mise à l’échelle de la PPrE doivent tenir compte de l’autoperception du risque de contracter le VIH et élargir l’accès aux dispensateurs de la PPrE.

## Introduction

Men who have sex with men (MSM) continue to see stable or increasing rates of HIV incidence worldwide (Beyrer et al. 2012). In 2014, 53% of all HIV infections in Canada occurred in MSM, and incidence was 131 times higher than among other men (Yang et al. 2016), emphasizing the need for new HIV prevention technologies. Pre-exposure prophylaxis (PrEP) with oral, daily tenofovir disoproxil fumarate/emtricitabine (TDF/FTC) was first shown to reduce HIV incidence through randomized placebo-controlled trials in 2010 (Okwundu 2012). Since then, studies among MSM have suggested real world reductions in HIV incidence of up to 100% among PrEP users in the United States and England (McCormack et al. 2016; Volk et al. 2015). As such, the Joint United Nations Programme on HIV/AIDS (UNAIDS) has identified PrEP as one of five pillars to achieve the goal of reducing incident HIV infections to less than 500,000 by 2020 (together with reproductive health services, evidence- and human rights-based prevention for key populations, condom programs and voluntary medical male circumcision) (UNAIDS 2016).

However, PrEP approval and uptake worldwide have been slower than expected. As of 2012, the only country which had formally approved PrEP was the US. Health authorities in France, South Africa, and Kenya followed in 2015, as did Canada in 2016 (Kirby and Thornber-Dunwell 2014; Cáceres et al. 2016). To optimize both public health impact and cost-effectiveness, PrEP should be targeted to those with highest HIV risk (Okwundu 2012; UNAIDS 2016). Understanding the barriers and facilitators of PrEP use in at-risk communities is important to achieve this goal. Yet there are few published data regarding levels of PrEP awareness, use, and interest among high-risk populations in Canada (Wilton et al. 2016; Escudero et al. 2015; Lebouche et al. 2016).

To inform local PrEP scale-up, we have previously quantified these parameters in a sample of MSM testing for HIV at clinic and community outreach sites in Toronto, Canada (Wilton et al. 2016). In this paper, our primary objective was to identify trends in the proportions of respondents who were aware of PrEP, were willing to use PrEP, and have ever used PrEP, using cross-sectional surveys administered at four time points between 2013 and 2016. Our secondary objectives were to assess the structural and personal barriers to PrEP use, and the key reasons for interest/disinterest in using PrEP in the most recent survey iteration.

## Methods

### Participants and setting

We distributed anonymous cross-sectional questionnaires to MSM presenting to Hassle Free Clinic and its satellite locations for anonymous point-of-care HIV testing at four successive time periods: (1) April–June 2013, (2) May–August 2014, (3) November 2014–April 2015, and (4) May–August 2016. Adult MSM who understood English were eligible to complete the survey immediately after their point-of-care HIV test or within 2 weeks if they returned to the study site. All participants were offered a CAD$10 gift card.

Hassle Free Clinic is a busy sexual health clinic in downtown Toronto that conducts approximately 5400 point-of-care HIV tests among MSM every year. Of these, over half are performed at four community-based satellite clinics across the city. Demographic characteristics and HIV positivity rates are generally similar across sites. Collectively, Hassle Free Clinic has the highest positivity rate (1.7%) of all 38 anonymous HIV testing sites in Ontario [Ken English, personal communication, March 2016].

### Survey instrument

Questionnaire content varied slightly between waves 1 and 4, but included demographic characteristics, sexual practices, recreational drug use, and perceived HIV risk, as well as awareness of, willingness to use, and current use of HIV prevention technologies such as post-exposure prophylaxis (PEP) and PrEP.

We quantified HIV risk using the HIV Incidence Risk Index for MSM (HIRI-MSM), a seven-item scale that incorporates (1) age, (2) number of male partners, (3) number of HIV-positive partners, (4) frequency of condomless receptive anal sex, (5) frequency of condomless insertive anal sex with HIV-positive partners, (6) amyl nitrate use, and (7) amphetamine use in the past 6 months (Smith et al. 2012). The scale generates scores ranging from 0 to 47, and a cutoff of ≥ 10 has been recommended for prioritizing men for intensive HIV prevention efforts such as PrEP. Because the sexual behaviour questions in survey wave 1 were worded slightly differently from the HIRI-MSM items, we imputed values for items 3–5 based on existing questions about the frequency of condom use, frequency of receptive anal sex, and frequency of insertive anal sex with HIV-positive partners. The same approach was used for item 3 in wave 2.

In the 2016 survey, we described PrEP as, “a new strategy for HIV prevention. It involves an HIV-negative person taking a pill DAILY, on an ongoing basis (starting before an exposure and continuing after for as long as the person is at risk) to reduce their risk of HIV infection. This pill contains two anti-HIV drugs (tenofovir/emtricitabine or Truvada®) and research suggests that it is relatively safe and is over 90% effective if taken consistently. It is much less effective if not taken every day and it does not protect against other STIs. Taking PrEP would require a visit to the doctor every 3 months in order to be tested for HIV, STIs and side effects. Truvada® was recently approved to be used for PrEP in Canada.” This description was modified for each wave of survey administration, in keeping with the approval status and available data on PrEP efficacy at the time. Each version was pilot-tested with five participants for clarity and face validity.

Awareness of PrEP was defined as responding “yes” to the question, “Before today, had you ever heard of PrEP?” Willingness to use PrEP was defined as responding with either “agree” or “strongly agree” on a five-point Likert-type scale to the statement, “I would be interested in taking PrEP to reduce my current risk of HIV infection”. Participants were also asked to indicate their reasons for being interested or not interested in PrEP, using a list of options derived from literature review and discussions with community members, including a free-text “other” field.

### Analysis

We summarized participant characteristics using descriptive statistics, with continuous variables summarized by measures of central tendency and dispersion and categorical variables by proportions. We used Kruskal-Wallis and chi-square tests to assess for differences in participant characteristics between survey waves.

To achieve our primary objective, we calculated the proportion of respondents in each survey wave who had each outcome of interest (awareness of PrEP, willingness to use PrEP, and ever having used PrEP), and conducted Cochran-Armitage Trend tests to assess for linear increasing trends in these proportions.

To further assess for temporal trends in our primary outcomes, we combined data from all survey waves and constructed multivariable logistic regression models to examine predictors of PrEP awareness and of willingness to use PrEP, using wave of survey administration as a continuous primary predictor variable. The low number of PrEP users precluded conducting regression analyses on PrEP use. Only variables present in all survey waves were considered as candidates for the multivariable models. When potential predictor variables were correlated (Spearman correlation or chi-square *p* < 0.01), only one was considered for inclusion in the multivariable model, based on model fit (AIC). We constructed models using forward selection, where variables were added one at a time and retained if statistically significant at a threshold of alpha = 0.10 (Maldonado and Greenland 1993).

Building on our previous work characterizing barriers to PrEP use in 2014–2015 (Wilton et al. 2016), we used data from the 2016 survey administration to construct hypothetical “PrEP cascades,” and quantified gaps between steps. To use PrEP, we reasoned that MSM must (a) be at risk of HIV infection, (b) be objectively high risk, (c) perceive themselves to be at moderate-to-high risk, (d) be aware of PrEP, (e) be willing to use PrEP, (f) have a family doctor, (g) be comfortable discussing sexual health with that doctor, and (h) have drug coverage or be willing to pay the full cost of the medications (CAD$850 per month) out of pocket. We selected the steps in the cascade based on clinical reasoning. For instance, all participants were anonymously testing for HIV and were therefore considered part of population A. Objective high risk was defined as a HIRI-MSM score ≥ 10. Perceived elevated risk was defined as indicating “medium or high risk” to the question, “What do you think your risk of getting HIV in the next year is?”

We assessed four hypothetical cascades (ABCDEFGH, ABDEFGH, ABCDEH, ABDEH) and analyzed them visually for gaps. We repeated this process using a higher HIRI-MSM score of ≥ 25 for defining high risk, as suggested by our prior work and by cost-effectiveness analyses (Wilton et al. 2016; MacFadden et al. 2016).

Finally, we tabulated self-identified barriers to using PrEP, and reasons for PrEP interest or non-interest in three key categories of respondents: high-risk individuals willing to use PrEP, high-risk individuals not willing to use PrEP, and low-risk individuals willing to use PrEP.

### Sample size

The target sample size for each wave was 400 responses, based on the number of respondents needed to estimate the primary outcome of interest (proportion with high perceived HIV risk and willing to use PrEP) with reasonable precision, as previously described (Wilton et al. 2016).

### Ethics approval

Prior to initiation, Research Ethics Boards of the University of Toronto, Ryerson University, and St. Michael’s Hospital approved the study. All potential participants were given a letter of information about the study to be read before beginning the survey, and survey completion constituted implied consent. All participants received counseling about HIV/STI testing, treatment, and risk reduction in the context of their clinical care.

## Results

Participant characteristics were generally similar for each of the four survey administrations (Table 1). Median (interquartile range, IQR) age was 31.0 (26, 38) years and most participants were white (54.0–59.5%), employed full time (67.8–72.0%), and college or university-educated (85.9–88.4%). Although some differences were observed between survey waves in the frequency of crack cocaine use, prior chlamydia and genital wart diagnoses, HIRI-MSM score, number of sexual partners, number of HIV-positive partners, perceived levels of HIV risk, and concern about HIV risk, most differences were modest.

**Table 1:** Participant characteristics

Characteristics	Survey wave 1 April–June 2013 (*n* = 436)	Survey wave 2 May–August 2014 (*n* = 400)	Survey wave 3 November 2014–April 2015 (*n* = 420)	Survey wave 4 May–August 2016 (*n* = 400)	*p* value
Age in years*	30 (26, 39)	31 (26, 38)	31 (26, 38)	30 (25, 38)	0.61^‡^
Ethnicity					
White^†^	251 (59.5)	216 (54.8)	230 (55.2)	216 (54)	0.20^§^
South Asian^†^	22 (5.2)	26 (6.6)	26 (6.2)	25 (6.3)	
Latino/Hispanic^†^	30 (7.1)	21 (5.3)	43 (10.3)	36 (9.0)	
Middle Eastern^†^	18 (4.3)	16 (4.1)	24 (5.8)	20 (5.0)	
Black^†^	19 (4.5)	19 (4.8)	16 (3.8)	16 (4.0)	
East Asian^†^	54 (12.8)	65 (16.5)	60 (14.4)	71 (17.8)	
Other/mixed^†^	28 (6.6)	31 (7.9)	18 (4.3)	16 (4.0)	
Education^**†**^					
High school diploma or less^†^	58 (13.7)	50 (12.6)	48 (11.5)	56 (14.1)	0.28^§^
College/university degree^†^	250 (59.0)	232 (58.6)	251 (60.3)	207 (52.1)	
Professional or graduate degree^†^	116 (27.4)	114 (28.8)	117 (28.1)	134 (33.8)	
Employment					
Full time^†^	286 (67.8)	285 (72.0)	290 (69.7)	284 (71.0)	0.29^§^
Part-time^†^	67 (15.9)	44 (11.1)	69 (16.6)	58 (14.5)	
Unemployed^†^	69 (16.4)	67 (16.9)	57 (13.7)	58 (14.5)	
Substance use in past 6 months					
Methamphetamines^†^	23 (5.5)	23 (5.8)	32 (7.7)	27 (6.8)	0.55^§^
Alcohol^†^	328 (77.7)	314 (79.5)	317 (76.4)	324 (81.0)	0.40^§^
Other recreational drugs^†^	61 (14.5)	62 (15.7)	46 (11.1)	62 (15.5)	0.20^§^
Poppers (amyl nitrates)^†^	126 (29.9)	104 (26.3)	123 (29.6)	128 (32.0)	0.37^§^
Crack cocaine^†^	19 (4.5)	57 (14.4)	58 (14.0)	78 (19.5)	< 0.0001^§^
Marijuana^†^	175 (41.5)	167 (42.3)	178 (42.9)	192 (48.0)	0.23^§^
None^†^	62 (14.7)	53 (13.4)	63 (15.2)	45(11.3)	0.36^§^
Lifetime diagnosis of STIs					
Gonorrhea^†^	–	96 (24.3)	87 (20.9)	106 (26.8)	0.14^§^
Chlamydia^†^	–	61 (15.4)	70 (16.8)	91 (23.0)	0.01^§^
Syphilis^†^	–	29 (7.3)	27 (6.5)	41 (10.4)	0.10^§^
Genital herpes^†^	–	16 (4.1)	18 (4.3)	16 (4.1)	0.97^§^
Genital warts^†^	95 (23.2)	58 (14.7)	65 (15.6)	60 (15.2)	0.003^§^
HIRI-MSM					
Score*	11 (8, 15)	12 (8, 19)	15 (8, 19)	15 (8, 21)	< 0.0001^‡^
≥ 10^†^	191 (50.7)	240 (62.2)	250 (64.4)	265 (66.4)	< 0.0001^§^
≥ 25^†^	16 (4.2)	47 (12.2)	55 (14.2)	69 (17.3)	< 0.0001^§^
Sexual behaviours					
Number of partners*	4 (2, 10)	5 (2,10)	5 (2, 10)	6 (3, 10)	0.0007^‡^
Number of HIV-positive partners^||^	0 (0, 1)	0 (0, 1)	0 (0,10)	0 (0,50)	< 0.0001^‡^
Number of unprotected anal receptive intercourse with any HIV status partner(s)*	0 (0, 0.5)	0 (0, 2)	0 (0, 2)	0 (0, 2)	0.02^‡^
Number of unprotected anal receptive intercourse with HIV-positive partner*	0 (0, 0)	0 (0, 0)	0 (0, 0)	0 (0, 0)	0.54^‡^
Perceived high HIV risk^†^	73 (17.3)	102 (25.9)	113 (27.2)	37 (9.3)	< 0.0001^§^
Concern about HIV risk^†^	189 (44.9)	141 (35.8)	157 (37.8)	80 (20.4)	< 0.0001^§^

*Median, (IQR)

^†^Frequency (%)

^‡^Kruskal-Wallis test

^§^Chi-square test

^||^Median, (range)

**Fig. 1:**
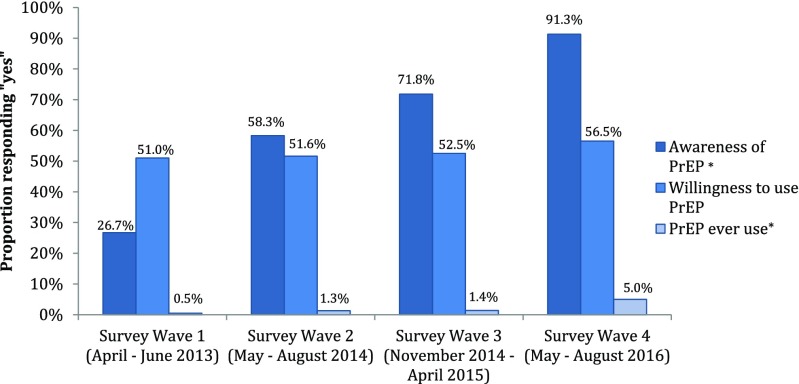
Trends in awareness of, willingness to use, and ever use of PrEP (*p < 0.0001)

Awareness of PrEP increased significantly over the four time periods from 26.7% (95% CI = 22.5–31.2%) to 58.3% (95% CI = 53.3–63.3%) to 71.8% (95% CI = 67.2–76.1%) to 91.3% (95% CI = 88.0–93.8%) (*p* < 0.0001, Fig. 1). Willingness to use PrEP may have increased slightly, from 51.0% (95% CI = 46.0–55.9%) to 51.6% (95% CI = 46.4–56.6%) to 52.5% (95% CI = 47.5–57.4%) to 56.5% (95% CI = 51.5–61.4%), but did not reach statistical significance (*p* = 0.06, Fig. 1). The frequency of ever having used PrEP was low but increased across all waves, at 0.5% (95% CI = 0.1–1.7%), 1.3% (95% CI = 0.4–3.0%), 1.4% (95% CI = 0.5–3.2%), and 5.0% (95% CI = 3.1–7.6%), respectively (*p* < 0.0001, Fig. 1). In the 2016 survey, current PrEP use was 3.0% (95% CI = 1.6–5.2%).

Variables associated with awareness of PrEP in multivariable logistic regression analyses are shown in Table 2. When attempting to build a multivariable model, survey wave was the only variable associated with PrEP awareness (OR = 2.83 per successive wave, 95% CI = 2.52–3.17). Other variables associated with PrEP awareness in univariable analyses were awareness of PEP (OR = 15.29, 95%CI = 11.86–19.72) and prior use of PEP (OR = 4.47, 95%CI = 2.66–7.53). In contrast, being concerned about HIV risk was associated with lower odds of PrEP awareness (OR = 0.71, 95% CI = 0.57–0.87).

**Table 2:** Associations with awareness of and willingness to use PrEP

	Awareness of PrEP				Willingness to use PrEP			
	Univariable		Multivariable*		Univariable		Multivariable	
	OR (95% CI)	*p* value	OR (95% CI)	*p* value	OR (95% CI)	*p* value	OR (95% CI)	*p* value
Time*	2.83 (2.52, 3.17)	< 0.0001	2.83 (2.52, 3.17)	< 0.0001	1.07 (0.98, 1.17)	0.12	1.04 (0.95, 1.14)	0.41
Age (by decade)	0.97 (0.88, 1.07)	0.52			0.92 (0.84, 1.02)	0.11		
Ethnicity								
White	1.00				1.00			
Black	0.94 (0.57, 1.57)	0.82			0.92 (0.56, 1.50)	0.74		
East Asian	0.66 (0.50, 0.88)	0.005			1.27 (0.96, 1.69)	0.10		
Latino/Hispanic	0.56 (0.39, 0.82)	0.003			1.39 (0.95, 2.03)	0.09		
Middle Eastern	0.99 (0.61, 1.61)	0.97			1.43 (0.88, 2.30)	0.15		
South Asian	0.76 (0.50, 1.17)	0.22			1.56 (1.02, 2.39)	0.04		
Other/Mixed	0.80 (0.51, 1.24)	0.31			1.43 (0.92, 2.21)	0.11		
Education								
College/university	1.00				1.00			
High school	0.71 (0.53, 0.96)	0.02			1.40 (1.04, 1.89)	0.03		
Employed full time	1.30 (1.04, 1.61)	0.02			1.04 (0.84, 1.29)	0.70		
Has private drug coverage	1.26 (1.03, 1.55)	0.03			0.91 (0.74, 1.11)	0.34		
HIRI-MSM score (per 10-point increment)	1.79 (1.56, 2.07)	<0.0001			1.81 (1.58, 2.07)	< 0.0001	1.61 (1.40, 1.86)	< 0.0001
Perceives moderate-to-high HIV risk	1.19 (0.92, 1.53)	0.18			3.25 (2.47, 4.27)	< 0.0001	2.44 (1.82, 3.27)	< 0.0001
Concerned about HIV risk	0.71 (0.57, 0.87)	0.001			2.85 (2.30, 3.54)	< 0.0001		
Aware of PEP	15.29 (11.86, 19.72)	< 0.0001			1.03 (0.84, 1.27)	0.78		
Prior use of PEP	4.47 (2.66, 7.53)	< 0.0001			1.68 (1.16, 2.44)	0.007	1.50 (0.998, 2.250)	0.051
Number of times of PEP use	1.34 (1.05, 1.70)	0.02			1.34 (1.05, 1.70)	0.01		

*When conducting multivariable analyses for the association between time and awareness of PrEP, time remained the only significant predictor

Variables associated with willingness to use PrEP in logistic regression analyses are shown in Table 2. Time was not a significant predictor in either univariable (OR = 1.07, 95% CI = 0.98–1.17) or multivariable models, with aOR = 1.04 (95% CI = 0.95–1.14). Consistent with our findings from wave 3 (Wilton et al. 2016), the only variables associated with willingness to use PrEP in adjusted analyses were moderate-to-high perceived HIV risk (aOR = 2.44, 95% CI = 1.82–3.27) and HIRI-MSM score (aOR = 1.61 per 10-point increase, 95% CI = 1.40–1.86); prior use of PEP had an aOR = 1.50 (95% CI = 0.998–2.250).

**Fig. 2:**
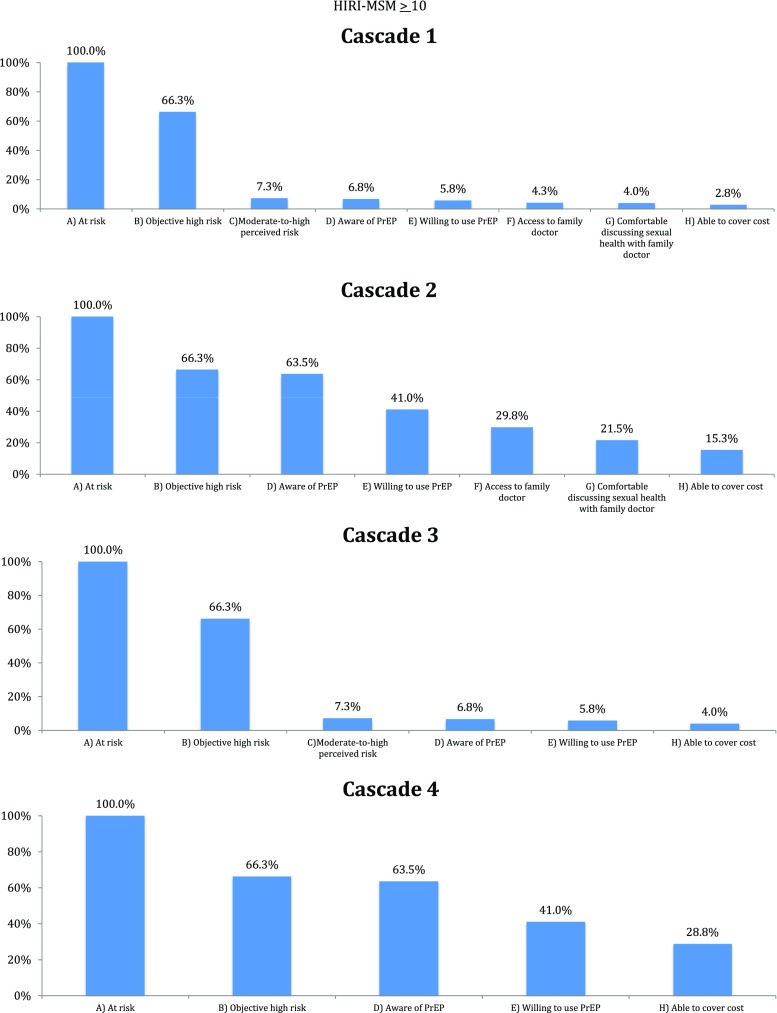

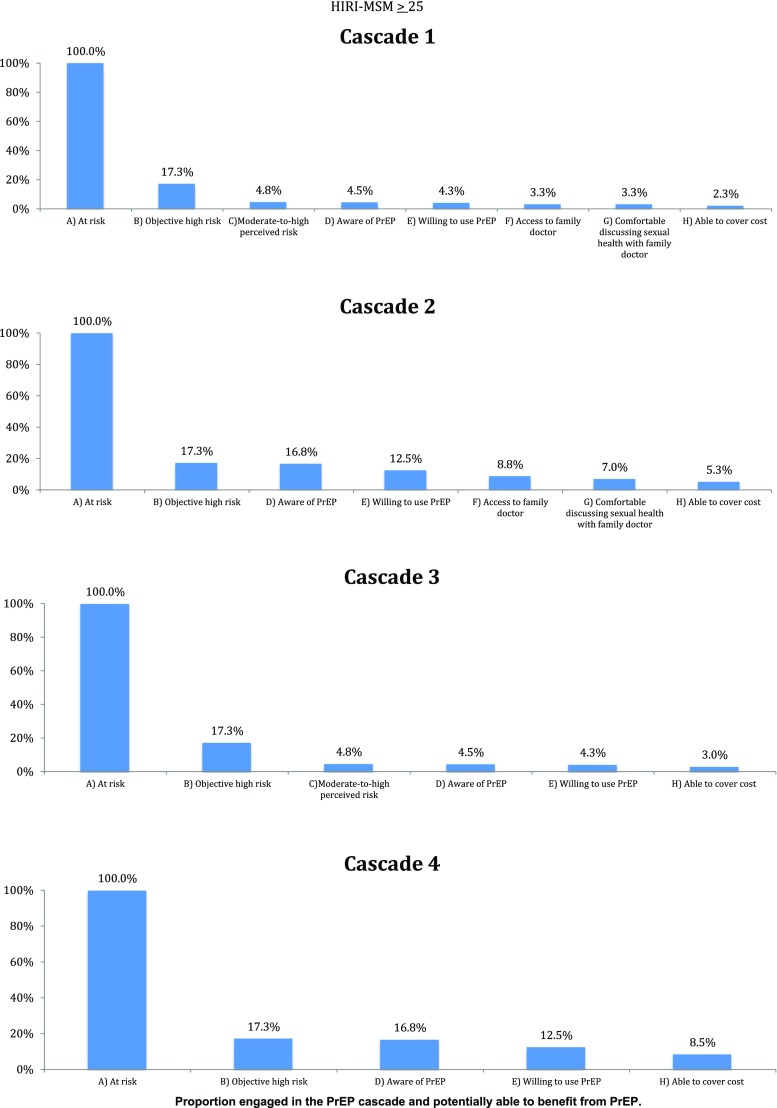
Proportion engaged in the PrEP cascade and potentially able to benefit from PrEP

The full cascade (ABCDEFGH) suggested that only 2.8% (11/400) participants had no individual or system barriers to using PrEP, similar to our previous findings (Fig. 2, cascade 1) (Wilton et al. 2016). The greatest barrier was inaccurate perception of HIV risk, as only 37 participants overall and 11.0% of those at elevated HIV risk (29/264) perceived their risk to be moderate-to-high. Removing moderate-to-high perceived risk from the cascade (ABDEFGH) improved hypothetical PrEP use to 15.3% (61/400) (Fig. 2, cascade 2). Willingness to use PrEP was the second largest barrier (56.5%, 226/400), followed by having a family doctor with whom respondents were comfortable discussing sexual health (53.8%, 214/400). To explore the potential impact of increasing the availability of PrEP providers, we removed the two steps relating to having a family doctor (ABCDEH) and found that the number of individuals who could use PrEP was still low at 4.0% (16/400). Jointly removing the self-perceived risk and PrEP provider steps (ABDEH) improved hypothetical PrEP use more substantially to 28.8% (115/400) (Fig. 2, cascade 4). A sensitivity analysis using a higher HIRI-MSM score of ≥ 25 to define high HIV risk resulted in a smaller proportion of participants who could use PrEP for each cascade (Fig. 2).

Since MSM at high objective HIV risk (HIRI-MSM ≥ 10) who indicate willingness to use PrEP represent an important group that should be prioritized for PrEP initiation, we tabulated perceived barriers to PrEP initiation in this group. While the most common barriers were related to financial access, including not having private drug insurance (33.1%, 95% CI = 25.8–41.1%) or believing that PrEP was not covered by one’s private insurance (34.4%, 95% CI = 27.0–42.4%), other common concerns included simply not having looked into getting PrEP yet (24.2%, 95% CI = 17.7–31.7%), not having a doctor (15.9%, 95% CI = 10.6–22.6%), and not feeling comfortable asking doctors for PrEP (14.0%, 95% CI = 9.0–20.4%), suggesting that efforts to increase the availability of trained PrEP providers and more actively facilitate linkages with PrEP services could increase uptake.

Those with high objective HIV risk who were unwilling to use PrEP are another group whose perspectives are important to understand (Table 3). Common reasons for their not being interested included being concerned about side effects (68.4%, 95% CI = 51.3–82.5%), believing they were not high risk enough (60.5%, 95% CI = 43.4–76.0%), the lack of 100% effectiveness (18.4%, 95% CI = 7.7–34.3%), and not trusting the science (7.9%, 95% CI = 1.7–21.4%). These findings suggest that interventions aimed at improving risk self-assessment and providing trustworthy information about PrEP’s proven safety and efficacy could be helpful.

**Table 3:** Reasons for wanting or not wanting PrEP by objective HIV risk

		Willing to use PrEP	
		Yes	No
Objective high risk	High	Group A (*n* = 169)	Group B (*n* = 38)
		• To decrease HIV risk (95.3%) • To decrease anxiety about getting HIV (69.8%) • To increase sexual pleasure by having condomless sex (45.6%) • To increase intimacy by having condomless sex (39.6%) • HIV prevention (3.6%) • Partner(s)’ pressure (1.8%)	• Concerned about side effects (68.4%) • Not high risk enough (60.5%) • PrEP not 100% effective (18.4%) • Cost (15.8%) • Do not want to visit doctor every 3 months (10.5%) • Other^a^ (13.2%) • Do not trust the science (7.9%)
	Low	Group C (*n* = 54)	
		• To decrease HIV risk (90.7%) • To decrease anxiety about getting HIV (81.5%) • To increase sexual pleasure by having condomless sex (16.7%) • To increase intimacy by having condomless sex (20. %) • Partner(s)’ pressure (1.9%)	

^a^Other includes: not interested in medications, possibility of risk compensation

Finally, we examined reasons for PrEP interest among those not at high objective HIV risk who nevertheless were willing to use PrEP (Table 3). Overall, this group had similar reasons compared to the “high risk, willing to use” group, but was numerically more likely to cite decreased anxiety about getting HIV (69.8 vs. 81.5%, *p* = 0.09) and less likely to cite interest in condomless sex as reasons (45.6 vs.16.7%, *p* = 0.0001 and 39.6 vs. 20.4%, *p* = 0.001).

## Discussion

In this series of four surveys among Toronto MSM undergoing anonymous HIV testing during the period 2013–2016, we observed a significant increase in PrEP awareness over time, reaching 91.3% in 2016. Although PEP awareness, PEP use and higher objective HIV risk were predictors of PrEP awareness in univariable analyses, time remained the strongest independent predictor when building the multivariable model. This association with time did not, however, correspond to a significant increase in willingness to use PrEP over time, which was 56.5% in 2016. In addition, PrEP uptake was low, with only 5.0% in the most recent survey reporting ever using PrEP.

The high degree of PrEP awareness in our 2016 sample is striking, and given the lack of concerted public health messaging about PrEP in Canada, is testament to the capacity of communities and healthcare providers to raise awareness about important health interventions at a population level. Conduct of Canada’s first PrEP demonstration project in the city from 2014 to 2016 (Tan et al. 2016), Health Canada approval of PrEP in February 2016 and information campaigns launched by local organizations may also have contributed to this increase. Our finding of 56.5% willingness to use PrEP is similar to that of a recent systematic review, which found overall PrEP acceptability to be 57.8% (95% CI = 52.4–63.1%) among MSM in 68 studies worldwide (Peng et al. 2017). Together with younger age, higher educational attainment, and wealth, that review also observed that prior awareness of PrEP was associated with higher acceptance (aOR = 1.33, 95% CI = 1.33–3.30) (Peng et al. 2017). Surprisingly, being concerned about HIV risk was negatively associated with PrEP awareness in our study, suggesting that greater efforts are needed to reach potentially interested individuals.

That willingness to use PrEP was stable despite rising PrEP awareness in this sample is similar to findings on MSM from Australia (Holt et al. 2017) and the USA (Grov et al. 2016), and could have several potential explanations. Awareness could be rising based on negative or inaccurate information about PrEP, which would suggest a greater need for public health authorities to provide an accurate and trusted voice. Alternatively, there may simply be a saturation point for willingness to use PrEP, which may be reassuring to those fearing that the resources required to meet demand for PrEP may be limitless.

From a public health perspective, however, the key consideration is how well PrEP awareness and willingness correspond to HIV risk. In this regard, it is reassuring that self-perceived HIV risk and higher objective HIV risk were independent predictors of willingness to use PrEP in our sample. Yet many of our respondents at elevated risk of HIV infection were unwilling to use it. Further, many respondents who were at high risk and willing to use PrEP were simply unable to access it. We further explored these challenges to PrEP implementation in two ways.

First, analogous to the concept of the HIV treatment cascade, previous studies have suggested actual or hypothetical PrEP cascades to identify the individual- and system-level factors that impede PrEP use (Wilton et al. 2016; Kelley et al. 2015). In our full cascade, the greatest barrier to using PrEP was low self-perceived HIV risk. Indeed, of those who were high risk and not willing to use PrEP in our study, over half believed they were not high risk enough. This complex relationship between actual and perceived risk has been observed elsewhere (Wilton et al. 2016; Moore et al. 2012). For instance, a study among HIV-negative Toronto MSM found that perceived risk increased when condom use with HIV-positive partners decreased, but found no association between risk perception and condom use in regular unknown HIV status partners (Kesler et al. 2016). Effective interventions to help at-risk individuals better understand and act on their HIV risk are available, and require broader uptake (reviewed in (HIV/AIDS 2017)). For example, in the THINK TWICE intervention, participants develop risk-reduction plans by working with a counselor to shape accurate beliefs and perceived risk through graphic novels and creating visual sexual network diagrams (HIV/AIDS 2017; Eaton et al. 2011). A combined biomedical/behavioural approach may have a greater impact on the epidemic by increasing awareness of risk as well as uptake of available HIV prevention interventions by those most at risk (Sullivan et al. 2012).

Second, we asked respondents to identify their own barriers to PrEP use, and found that apprehensions about side effects and about the imperfect efficacy of PrEP were the predominant concerns. These factors likely underlie our finding that lack of willingness to use PrEP was the second most common barrier in our cascade analyses. To address these barriers, public health authorities and other stakeholders should publicize the well-established safety and efficacy of PrEP (Fonner et al. 2016) and emphasize that true “breakthrough infections” are extremely rare when PrEP adherence is high (Knox et al. 2017).

Finally, our cascades and questions about reasons for wanting/not wanting PrEP showed that the next most important barriers were access to PrEP providers and drug affordability. Over half of respondents lacked a family doctor with whom they felt comfortable discussing sexual health, emphasizing that public health bodies must train more providers to deliver PrEP in a non-stigmatizing way. Involving a broader range of healthcare professionals in PrEP delivery, including nurses, nurse practitioners and pharmacists, may also improve provider capacity (Cáceres et al. 2016; Sharma et al. 2017; Sawkin and Shah 2016). Further, given that high cost is a known impediment to PrEP use that may amplify health inequities (Kelley et al. 2015; Doblecki-Lewis et al. 2017), structural interventions and political action may be needed to prioritize access for those who are at greatest HIV risk (Cáceres et al. 2016).

Our study has limitations that warrant consideration. First, we recruited MSM seeking anonymous HIV testing at a facility with a high HIV diagnosis rate, and our sample was largely employed and well-educated. While these factors may limit the generalizability of our findings to all at-risk MSM, our sample is representative of the population served by our recruitment site, an important site for PrEP referrals and assessments in Toronto. Second, there were minor changes in the wording of some questions and in the description of PrEP between survey waves. While these differences may have impacted responses (e.g., mentioning Health Canada approval in the 2016 wave could have increased willingness to use PrEP), these changes reflect the changing contexts in which respondents formulated their opinions about PrEP, and were necessary to ensure that information provided to participants about PrEP was up-to-date. Wording changes also necessitated imputation of some values when calculating HIRI-MSM scores for survey waves 1 and 2; such changes may have contributed to the small observed differences in participant characteristics between waves but would not have affected our primary or secondary outcomes. Third, HIRI-MSM has limited sensitivity for identifying HIV seroconverters in some contexts, and thus may have underestimated the proportion of those who should use PrEP (Lancki et al. 2018). Further, it is possible that repeated administration of the survey may have itself contributed to increases in PrEP awareness in the study population. Finally, our analysis assumed that each completed survey over the four waves corresponded to a unique individual, although this could not be confirmed because surveys were completed anonymously.

## Conclusion

Our study identified increasing awareness and consistent, moderate willingness to use PrEP among at-risk Toronto MSM. To maximize the potential public health benefit or PrEP, next steps should include developing strategies for helping men better understand their HIV risk, publicizing the excellent safety and efficacy of PrEP, training providers for PrEP delivery, and identifying funding strategies to facilitate PrEP use.
